# A bounded hierarchy framework for the evolution of syntax

**DOI:** 10.1007/s10539-025-09998-w

**Published:** 2025-10-13

**Authors:** Giulia Palazzolo

**Affiliations:** Department of Philosophy, University of Warwick, Coventry, UK

**Keywords:** The evolution of human syntax, Animal sequences, The merge hypothesis, Hierarchy, Compositional semantics, Language evolution

## Abstract

Is syntax an evolutionary novelty in the human lineage? This question, along with the question of how human syntax evolved, is highly debated in the field of language evolution. In this paper, I reconstruct two prominent frameworks for studying the evolution of human syntax, which I call “unbounded hierarchy” (Bolhuis et al. 2018 in PLoS Biol 16(6):e2005157, 2018. 10.1371/journal.pbio.2005157) and “compositional semantics” (Townsend et al. 2018 in PLoS Biol 16(8):e2006425, 2018. 10.1371/journal.pbio.2006425). I argue that both frameworks face problems when it comes to explaining the evolution of human syntax. Considering these problems, as well as empirical evidence of hierarchy in nonhuman animals, I provide an alternative framework for studying the evolution of human syntax, which I call “bounded hierarchy”. The bounded hierarchy framework that I propose traces the evolutionary origins of human syntax to simpler forms of bounded hierarchy that may be present in extant nonhuman animals.

## Introduction

Natural languages are composed of atomic elements, like words, that can be combined into sequences, like sentences, to convey different meanings. This property of natural languages is commonly called syntax. The capacity for syntax—that is, the ability to combine linguistic units into structured sequences—enables humans to express an astonishing variety of different thoughts using just a limited set of elements.

The question of how human syntax evolved is highly debated in the field of language evolution. Is syntax an evolutionary novelty in the human lineage? Or can precursors be found in the capacities of other species? According to an influential view, syntax is a uniquely human capacity, which is the product of a sudden genetic mutation that marks a substantial evolutionary gap between humans and other animals (Berwick and Chomsky 2016; Chomsky et al. 2023). However, recent evidence from comparative studies suggests that some nonhuman animals also combine their signals in larger sequences. This increasing evidence has been found in a wide range of animal species, including primates, birds and cetaceans (e.g. putty-nosed monkeys, Campbell’s monkeys, chimpanzees, bonobos, see e.g. Arnold and Zuberbühler 2012, Ouattara et al. 2009, Leroux et al. 2023, Berthet et al. 2025; Japanese tits, southern pied babblers, Bengalese finches, nightingales, see e.g. Suzuki et al. 2018, Engesser et al. 2016, Berwick et al. 2011; whales, see e.g. Mercado and Perazio 2021, Sharma et al. 2024). The evidence of animal sequences has begun to challenge the claim that syntax is uniquely human. Drawing on this evidence, Townsend et al. (2018) have argued that syntax could have evolved from certain combinatorial aspects of the communication systems of other animals.

Starting from the debate between Bolhuis et al. (2018) and Townsend et al. (2018) on the case study of Japanese tits ‘ABC-D’ calls (Suzuki et al. 2018), in this paper I reconstruct two prominent frameworks for studying the evolution of human syntax, which I call “unbounded hierarchy” (Bolhuis et al. 2018) and “compositional semantics” (Townsend et al. 2018). These frameworks identify different requirements for syntactic continuity and provide different accounts of the evolution of human syntax. The unbounded hierarchy framework argues that the animal sequences studied so far are not continuous with human syntax. The compositional semantics framework sets looser requirements for syntactic continuity that can be met by some animal sequences. As a result, the unbounded hierarchy framework provides a saltationist account of the evolution of human syntax, according to which syntax evolved all of a sudden and exclusively in the human lineage. The compositional semantics framework provides an incrementalist account, according to which human syntax could have evolved from certain animal capacities.

I argue that both frameworks face problems when it comes to explaining the evolution of human syntax. Considering these problems, as well as empirical evidence of hierarchy in nonhuman animals, I provide an alternative framework for studying the evolution of human syntax, which I call “bounded hierarchy”. The bounded hierarchy framework that I propose traces the evolutionary origins of human syntax to simpler forms of bounded hierarchy that may be present in extant nonhuman animals.

Here is the structure of this paper. “The debate between Bolhuis et al. (2018) and Townsend et al. (2018) on the case study of Japanese tits ‘ABC-D’ calls” section introduces the debate between Bolhuis et al. (2018) and Townsend et al. (2018) on the case-study of Japanese tits’ ‘ABC-D’ calls. “The unbounded hierarchy framework for the study of the evolution of human syntax” section presents the unbounded hierarchy framework for the study of the evolution of human syntax. “The compositional semantics framework for the study of the evolution of human syntax” section presents the compositional semantics framework. “Two accounts of the evolution of human syntax” section discusses two accounts of the evolution of human syntax associated with these frameworks. The problems of the unbounded hierarchy and compositional semantics frameworks are discussed in “Problems with the unbounded hierarchy framework” and “Problems with the compositional semantics framework” sections, respectively. In “Toward a bounded hierarchy framework” and “Vindicating the possibility of a bounded hierarchy” sections, I make a case for my bounded hierarchy framework for the study of the evolution of human syntax on the basis of both theoretical reasons and empirical evidence.

## The debate between Bolhuis et al. (2018) and Townsend et al. (2018) on the case study of Japanese tits ‘ABC-D’ calls

It is Suzuki et al.’s contention in their paper of 2018 that the evidence gathered on Japanese tits suggests that they possess human like syntactic abilities, specifically compositional syntax. Like human syntax, Suzuki et al. (2018: 2) argue, the ‘ABC-D’ sequences of Japanese tits seem to follow ‘the principle of compositionality’, in which the meaning of a combination “depends on both the meaning of its parts and the way in which they are combined”.

According to Suzuki et al. (2018), three conditions are required to establish compositional syntax in nonhuman animals:

(i) The production of call sequences is tied to a specific context.
(ii) Receivers respond to the sequence by interpreting the meanings of the component calls.
(iii) The response to the sequence is tied to the way in which the component calls are combined.

Condition (i) ensures that the call sequence is meaningful––that is, it conveys specific information. Condition (ii) ensures that the sequence functions as a combination of independently meaningful signals, rather than as a single, acoustically complex, meaningful unit (cf. Schlenker et al. 2023’s ‘one-utterance’ hypothesis^1^). Condition (iii) assesses whether, similar to natural language speakers, nonhuman animals are sensitive to the order in which calls appear in the sequence. In Suzuki et al. (2018)’s view, the ‘ABC-D’ calls of Japanese tits satisfy all three conditions:

(i) The ‘ABC-D’ sequence is consistently produced in mobbing situations––situations in which Japanese tits seek to recruit other flock members to mob a predator who is threatening their nest. The meaning of the sequence is thought to be something like [/Threat! Come here!/].
(ii) Receivers’ responses suggest that they interpret the sequence by deriving the individual meanings of ‘ABC’ and ‘D’ calls. In Japanese tits, ‘ABC’ calls are general alert calls produced to warn conspecifics of potential predators (like saying [/threat!/]). ‘D’ calls are recruitment calls and are used to attract conspecifics (like saying [/come here!/]). Scanning the surrounding and approaching the sender are the typical responses to ‘ABC’ and ‘D’ calls, respectively. In response to ‘ABC-D’ calls, Japanese tits approach the sender while continuously scanning their surroundings for threats. Another experiment by Suzuki et al. (2017), featuring Willow tits’ ‘tää’ calls, further rules out the possibility that the ‘ABC-D’ calls constitute a single, acoustically complex, signal. ‘Tää’ calls, like ‘D’ calls in Japanese tits, are recruitment calls. In areas where Willow tits and Japanese tits forage together, Japanese tits recognise and respond to tää calls in a similar way to their own species-specific ‘D’ calls, i.e., by approaching the caller. In their study, Suzuki et al. (2017) present Japanese tits with artificially constructed ‘ABC-tää’ sequences—sequences in which their natural ‘D’ calls have been replaced by the corresponding ‘tää’ calls of Willow tits. The results of this experiment show that, presented with artificially constructed ‘ABC-tää’ sequences, Japanese tits respond as they do when they receive their own ‘ABC-D’ sequences: they scan the environment in search for possible threats while approaching the caller (in this case, the loudspeaker). Japanese tits’ response to ‘ABC-tää’ sequences suggests that they treat ‘ABC-D’ as a sequence of two individual signals, rather than as a single signal (the one-utterance hypothesis). If they treated ‘ABC-D’ as a single utterance, we should not expect them to respond to ‘ABC-tää’ at all, even if they know the meaning of ‘tää’. ‘ABC-tää’ would in fact be a different call from ‘ABC-D’. Note that the ‘tää’ calls of Willow tits are also acoustically distinct from Japanese tits’ ‘D’ calls. This undermines the possibility that Japanese tits respond to ‘ABC-tää’ and ‘ABC-D’ alike just because these sequences are acoustically similar.
(iii) Japanese tits exhibit reduced responses to artificially reversed ‘D-ABC’ sequences, which might indicate that they are sensitive to the order of call units. In Suzuki et al.’s view, Japanese tits could perceive the meaning [/Come here! Threat!/] as more ambiguous than [/Threat! Come here!/].

On this basis, Suzuki et al. (2018) argue that, contrary to prevalent views, the principle of compositionality may not be unique to human language. They suggest that investigating compositionality in other animal communication systems may provide valuable insights into the evolution of human syntax.

The study by Suzuki et al. of the ‘ABC-D’ calls of Japanese tits has attracted conflicting interpretations from two groups of scholars: Bolhuis et al. (2018) and Townsend et al. (2018). On the one hand, Bolhuis et al. (2018) deny that the ‘ABC-D’ calls are in any respects comparable to human syntax. This is for two reasons. First, Japanese tits do not appear to freely combine their calls, as language speakers do. Bolhuis et al. point out that Japanese tits’ sequences are very limited in both number and range. There is no evidence that Japanese tits produce longer sequences than ‘ABC-D’ or that they produce different combinations. For example, there is no evidence that they combine food and recruitment calls to invite conspecifics to share food, just as they combine alarm and recruitment calls to invite conspecifics to mob. Second, there is no evidence that these sequences share the hierarchical structure of language. Bolhuis et al. are careful to point out that hierarchical structure, and not linear order, plays a central role in human syntax. Even if we accept that Japanese tits are sensitive to linear order, this is not enough to attribute to them a human-like compositionality.^2^

On the other hand, Townsend et al. (2018) argue that there is more continuity between compositionality in animals and humans than Bolhuis et al. are willing to admit. According to Townsend et al., the absence of hierarchy and generativity in animal combinations does not pose a significant challenge for continuity claims, because human language also contains examples of non-hierarchical and non-generative structures that, they claim, are nonetheless intuitively syntactic. Townsend et al. illustrate their claim by providing examples of phrases like ‘duck and cover’, which they take to be non-hierarchical, and ‘gimme me a break’, which they take to be non-generative, since it is ordinarily not embedded into larger syntactic strings. Given the existence of non-hierarchical and non-generative structures in human language, Townsend et al. argue that animal sequences can be seen as exhibiting continuity with human syntax, even if they are non-hierarchical and non-generative.

The debate between Bolhuis et al. (2018) and Townsend et al. (2018) highlights several key points of disagreement about what is required for the study of the evolution of human syntax. Two frameworks can be extracted from this debate: these are what I call the framework of unbounded hierarchy and the framework of compositional semantics. The following two sections will expound on the core requirements of each framework.

## The unbounded hierarchy framework for the study of the evolution of human syntax

Within the framework of unbounded hierarchy, for animal sequences of signals to be regarded as continuous with human syntax, they must fulfil two basic requirements:

(UH.1) *Hierarchy:* The sequence must exhibit a hierarchical structure.

(UH.2) *Unboundedness:* The sequence must be the product of an unbounded combinatorial capacity, i.e. virtually infinite in both number and range of signals that can be combined.

Requirements UH.1 and UH.2 correspond to what, within the unbounded hierarchy framework, are considered to be two fundamental features of human syntax: hierarchy and unboundedness. I will discuss each of these features in turn.

Human syntax has hierarchy because linguistic expressions are not merely flat strings of words, but multi-layered constructions in which units function as constituents––parts of larger structures such as phrases and clauses. As Bloomfield (1933) introduces the constituent structure that characterises natural language syntax:

“Any English-speaking person who concerns himself with this matter, is sure to tell us that the immediate constituents of “Poor John ran away” are the two forms “poor John” and “ran away”; that each of these is, in turn, a complex form; that the immediate constituents of “ran away” are “ran” and “away”; and that the constituents of “poor John” are “poor” and “John”” (Bloomfield 1933 as reported in Lyons 1968).^3^

The analysis of a sentence like “Poor John ran away” into its several layers of constituents can be illustrated graphically in a number of ways. One is to use a bracket notation: [(Poor John) (ran away)]. Another is to construct a tree diagram, in which nodes represent higher-order structures into which lower-level units are embedded:
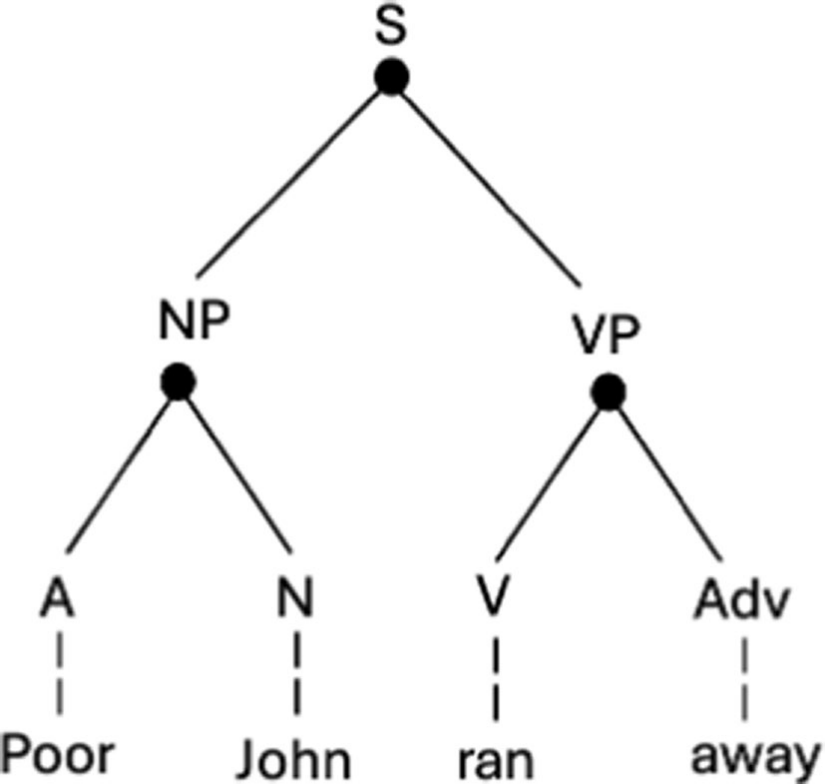


Human syntax is taken to have unboundedness in the sense of being the product of an unbounded combinatorial capacity, whose combinations of elements are virtually infinite. There are two main senses of unboundedness in the unbounded hierarchy framework. First, there is no principled limit to the number of elements that can be combined. For example, in natural languages many words can be combined with one another, to form long and complex sentences, without any determinate upper limit. Second, there is no principled limit to the range of combinations that can be formed. For example, in natural languages many different words can be combined together to create a vast, virtually infinite, range of different sentences.

Proponents of the unbounded hierarchy framework take human unbounded combinatorial capacity to be grounded in a specific cognitive operation called Merge (Berwick and Chomsky 2016; Chomsky et al. 2023). It is this cognitive operation that, for these scholars, makes human syntax hierarchical and unbounded. As Chomsky et al. (2023: 12) describe it, Merge is the simplest possible computational operation: it takes any two syntactic objects, combines them into sets, and it can recursively reapply to its own output. For example, Merge can combine “the” and “book” to create the set {the, book}, then combine that set with “bought” to form the structure {bought, {the, book}, and so on indefinitely (Berwick and Chomsky 2016: 10). By reapplying to its own output, Merge can generate structures of potentially unlimited hierarchical depth.^4^

Proponents of the unbounded hierarchy framework argue that the animal sequences that have been studied so far cannot be considered continuous with human syntax because they fail to satisfy requirements UH.1 and UH.2: neither they have hierarchy, nor are they the product of an unbounded combinatorial capacity (see e.g. Bolhuis et al. 2018). As we will see in “Two accounts of the evolution of human syntax” section, scepticism about animal syntax leads proponents of the unbounded hierarchy framework to advocate a saltationist account of the evolution of human syntax.

## The compositional semantics framework for the study of the evolution of human syntax

Within the framework of compositional semantics, for animal sequences of signals to be regarded as continuous with human syntax, they must fulfil two basic requirements:

(CS.1) The sequence must be composed of meaningful units.

(CS.2) The meaning of the sequence must be composed of the meanings of the individual signals.

Requirements CS.1 and CS.2 correspond to what, within the compositional semantics framework, are considered to be two fundamental features of human syntax. First, in human syntax sentences are composed of meaningful units (e.g. words). Second, the meaning of a sentence depends on the meanings of the units of which it is composed. Consider again the English sentence “Emma saw the pirate with binoculars”. The meaning of this sentence is composed of the meanings of the words “Emma”, “pirate” and “binoculars”.^5^

Certain animal sequences, such as the Japanese tits ‘ABC-D’ calls, satisfy requirements CS.1 and CS.2. As we saw in “The debate between Bolhuis et al. (2018) and Townsend et al. (2018) on the case study of Japanese tits ‘ABC-D’ calls” section, the ‘ABC-D’ sequences are composed of the individual meaningful elements ‘ABC’ and ‘D’ (CS.1); and receivers’ responses suggest that their meaning is composed of the meanings of ‘ABC’ ([/Threat!/] and ‘D’ ([/Come here!/], being something akin to [/Threat! Come here!] (CS.2). Thus, according to proponents of compositional semantics, these sequences can be regarded as significantly continuous with human syntax and can be relevant to understanding its evolution.

## Two accounts of the evolution of human syntax

Proponents of unbounded hierarchy and compositional semantics advocate two alternative accounts of the evolution of human syntax: one saltationist, the other incrementalist. According to the saltationist account advocated by proponents of the unbounded hierarchy framework, syntax evolved all of a sudden and exclusively in the human lineage. According to Berwick and Chomsky (2016), being Merge the simplest possible computational operation, it can be the result of an abrupt evolutionary event, like a genetic mutation bringing about a minor rewiring of the brain. Mukherji (2022: 86) describes the origin of syntax in terms of a ‘lightning strike’, to emphasise its immediate, saltationist character. Proponents of the Merge hypothesis place the event at the origins of syntax sometime between 200,000 years ago (when anatomically modern humans first appeared) and 60,000 years ago (the time of the exodus from Africa), most likely around 80,000 years ago, when some rapid behavioural changes occurred (Berwick and Chomsky 2016; Tattersall 2008; Jobling et al. 2014).^6^

Proponents of the compositional semantics framework, on the other hand, pursue the possibility of an incrementalist account of the evolution of human syntax, according to which human syntax evolved from certain combinatorial aspects of the communication systems of other animals––in particular, from those capacities displayed by nonhuman animals in processing sequences that exhibit a compositional semantics. Note that scholars like Townsend et al. (2018) remain neutral as to whether specific animal sequences are analogous or homologous features with respect to human syntax:

Whether or not comparable combinations across species are indeed evolutionarily related is an unresolved issue. […]. Only once these data are at hand can we feasibly start to empirically explore the phylogeny of syntax, e.g., whether alarm call combinations in monkeys are truly homologous to a conjunction of commands in humans or whether a bird alert–recruitment call combination is a genuine analogue of monkey alarm combinations or of human command conjunctions (Townsend et al. 2018: 3-4).

While homologous features are similar in form because they derive from a common ancestral trait (i.e. have a shared evolutionary history), analogous features are similar in form because, for example, they arose in response to similar environmental pressures but have evolved independently (Ruse and Travis 2009).

In the next two sections, I will assess the frameworks of unbounded hierarchy and compositional semantics in relation to their capacity to identify behaviours that are significantly continuous with human syntax and that can be relevant to understanding its evolution. As I will show, both frameworks are not without problems when it comes to explaining the evolution of human syntax. Importantly, I will not evaluate these frameworks in relation to their capacity to identify actual evolutionary precursors of human syntax in animal behaviours. Indeed, in this paper I remain neutral on the question of whether specific animal behaviours are analogous to, or homologous with, human syntax. While notable similarities between human syntax and some animal behaviours make the hypothesis of a common ancestry attractive, as things currently stand it is not possible to rule out that these behaviours emerged independently, through separate evolutionary histories. This possibility should be considered especially in those cases where such behaviours are found only in a few, phylogenetically distant species. Still, whether analogous or homologous features, identifying behaviours that exhibit a significant continuity with human syntax can illuminate important aspects of its evolution (for a similar point, see also Palazzolo 2024 and Palazzolo forthcoming): animal behaviours that are homologous can shed light on the phylogenetic origins of human syntax. Meanwhile, animal behaviours that are analogous can shed light on the kinds of environmental conditions that support the emergence of syntactic capacities.

## Problems with the unbounded hierarchy framework

There is broad consensus that hierarchy is a basic feature of human syntax: in human syntax, higher-level structures incorporate multiple, embedded, lower-level units (Jackendoff 2002, Progovac 2015; Bolhuis et al. 2018; Planer and Sterelny 2021; Coopmans et al. 2022; Planer 2023).^7^ Evidence for syntactic hierarchy in natural language can be found in different linguistic phenomena. An important case is the one of ambiguous utterances, where a same sequence of words can admit different interpretations depending on how elements are grouped together and contained within one another. For example, consider the English sentence “Emma saw the pirate with binoculars.” On one interpretation, Emma uses binoculars to see the pirate ([Emma [saw [the pirate]] with binoculars]); on another interpretation, the pirate is the one with binoculars ([Emma [saw [the pirate with binoculars]]]). The ambiguity arises from different possible hierarchical groupings of the sentence’s constituents.

Lidz et al. (2003) consider the structure-dependent interpretation of phenomena like anaphora another indicator of hierarchy in natural language. When presented with a sentence like “Look! A yellow bottle. Do you see another one?” language speakers as young as 18 months interpret the pronoun “one” as modifying the entire constituent “yellow bottle” rather than just “bottle”. This indicates that the elements “yellow” and “bottle” are processed as organised into the larger structure “yellow bottle” (Lidz et al. 2003). Empirical findings from Coopmans et al. (2023) suggest that speakers typically prefer hierarchical interpretations over linear interpretations of sentences. Hierarchy is also said to emerge spontaneously in cases of home sign, where children are not exposed to the input of conventional languages (Hunsicker and Goldin-Meadow 2012). Importantly, the structural relationships between words in natural language need not follow linear order. Dependencies, in other words, can be non-adjacent: as Berwick and Chomsky (2016) note, in a sentence like “instinctively birds that fly swim”, the adverb “instinctively” modifies “swim” and not “fly”, even though “fly” comes first in the linear sequence.

If hierarchy is a basic feature of natural language, the study of the evolution of human syntax should address the question of the evolution of hierarchy. Thus, Bolhuis et al. (2018) and the other proponents of the unbounded hierarchy framework are right in identifying hierarchy as a requirement for syntactic continuity (UH.1), and in looking for evidence of hierarchy in animal sequences.^8^ However, the unbounded hierarchy framework is unwarranted in establishing a discontinuity between animal sequences and human syntax on the basis of UH.2: animal sequences must be the product of an unbounded combinatorial capacity (Merge). The reason why requiring UH.2 is unwarranted becomes evident when we consider the distinction between competence and performance in the Merge hypothesis.

In the Merge hypothesis, the claim about unboundedness is not an empirical one; rather it is a claim about competence. Human combinatorial competence is unbounded in its capacity to combine elements (Chomsky 1980). Empirically, there are obvious practical limits to the human syntactic capacity. For example, speakers can process sentences only up to a certain length and complexity (Lakretz et al. 2020). According to proponents of the Merge hypothesis, this is because at the level of performance (i.e. actual language use), Merge must inevitably come to terms with the limits of the human cognitive system, such as limited memory and attention.

Proponents of the unbounded hierarchy framework claim that animal sequences do not satisfy UH.2 because they are bounded (i.e. limited in number and range of elements, Bolhuis et al. 2018). However, the claim that human syntax alone is the product of an unbounded combinatorial capacity turns on an illegitimate comparison between competence and performance (Moore and Palazzolo 2024).^9^ What animals do in practice (performance) is compared with what humans could do in principle (competence), if unrestrained by their limited cognitive resources. Moreover, on the one hand, proponents of the unbounded hierarchy framework are willing to concede that human syntactic capacity is bounded in practice (performance) but unbounded in principle (competence). On the other, when it comes to assessing animal syntactic capacity, they are not willing to do the same. On the contrary, they use evidence that animal syntactic capacity is bounded in practice (performance) to draw conclusions about what animals could do in principle (competence).

Even if there is little or no evidence of syntactic complexity in the animal kingdom, we should not draw the conclusion that unbounded competence (Merge) is uniquely human. If some animal sequences turn out to be hierarchically structured, even if they are bounded in practice (performance), they could potentially still be the result of a combinatorial capacity that is unbounded in principle (competences), though restrained by the limited cognitive resources of animals (e.g., because of limitations on working memory and attention—abilities that are very limited in many animal species).^10^

Not only is the unbounded hierarchy framework unwarranted in establishing a discontinuity between animal sequences and human syntax on the basis of UH.2, but it also seems unwarranted in requiring UH.2 for syntactic continuity. Proponents of unbounded hierarchy assume that precursors of an unbounded capacity cannot be found in bounded capacities. As Berwick and Chomsky (2016: 107) put it, “there can be no series of small steps that leads to infinite”. Just as something is either infinite or not, and there is no more or less to infinity, so too the infinite combinatorial capacity is either present or absent, with no intermediate stages in between. As argued by Berwick (1998: 338–9), “there is no possibility of an ‘intermediate’ syntax between a non-combinatorial one and full natural language—one either has Merge in all its generative glory, or one has no combinatorial syntax at all”.

However, there seems to be no principled reason why precursors of an unbounded capacity cannot be found in bounded capacities. For example, if we take the case of numerical cognition, on some views, such as Carey (2009)’s, our unbounded numerical capacities (e.g. the ability to count) have roots in core abilities to track finite quantities and come about only later, both ontogenetically and phylogenetically, through the acquisition of natural languages. Accounts of the evolution of syntax such as those by Jackendoff (2002) and Progovac (2015) show that human syntax itself could have plausibly evolved in multiple, progressive stages.

On these grounds, I argue that, requiring unboundedness (UH.2), the unbounded hierarchy framework sets too high of a bar for syntactic continuity. First, it requires empirical evidence of unbounded competence, which we do not possess even in the case of humans. Second, it is unjustified in ruling out that precursors of an unbounded capacity could be found in bounded capacities.

## Problems with the compositional semantics framework

In “Problems with the unbounded hierarchy framework” section, I argued that the unboundedness requirement (UH.2) of the unbounded hierarchy framework is unwarranted, and that this framework is unjustified in establishing a discontinuity between animal sequences and human syntax on the basis of this requirement. The compositional semantics framework takes seriously the possibility of an incrementalist account of the evolution of human syntax, but it does so at the cost of sacrificing the hierarchy requirement (UH.1). This creates two main problems for the compositional semantics framework. First, only requiring CS.1 and CS.2 is not enough to ensure that signals in the sequences are actually combined with one another. Second, in considering animal sequences that are not hierarchically structured continuous with human syntax, the compositional semantics framework does not have anything to say about the evolution of hierarchy, which is a basic feature of human syntax, one that should be accounted for in one form or another.

I will start from the first problem. Consider once again the case study of the ‘ABC-D’ calls in Japanese tits (Suzuki et al. 2018). Townsend et al. (2018)’s hypothesis that these sequences satisfy the requirements of compositional semantics (CS.1 and CS.2) is constructed from the observation that receivers respond simultaneously to ‘ABC’ and ‘D’ calls. The fact that Japanese tits adopt behavioural responses that are compatible with the presentation of both signals suggests that the sequence is composed of individually meaningful signals (CS.1) and that the receivers derive the meaning of the sequence from its parts (CS.2). However, this does not rule out the possibility that receivers respond to ‘ABC’ and ‘D’ calls separately. As Schlenker et al. (2016) note, each signal in the ‘ABC-D’ sequence may reflect an independent, internal state of the signaller at a specific time T (e.g., “threat at time T” and “help needed at time T”) and may be interpreted as such by the receivers. This would be a case of spatial–temporal juxtaposition of meaningful signals ‘ABC’ and ‘D’, as opposed to a genuine combination ‘ABC-D’ (see also Schlenker et al. 2023).

Furthermore, quantitative empirical criteria for identifying sequences of signals, such as temporal proximity and non-randomness (Suzuki et al. 2018), are insufficient to determine whether ‘ABC-D’ calls form genuine combinations. Signals may occur in rapid succession for reasons unrelated to syntax. For example, in the case of young chimpanzees’ synonymic gestural sequences, gestures occur in close temporal proximity, but these gestures are not combined with one another. As noted by Hobaiter and Byrne (2011), chimpanzees increase the chances of their gestures being received or responded to by rapidly producing synonymic gestures one after the other. Additionally, the fact that ‘ABC’ and ‘D’ signals occur together above chance (i.e., more often than random juxtaposition of single signals, Girard-Buttoz et al. 2022) does not guarantee that these signals form genuine combinations. The non-randomness of certain sequences could be explained by the fact that certain co-occurrences of mental states or intentions are simply more likely than others. For example, you may not get food and alarm calls together because it is unusual for an individual to intend to gather conspecifics around a food source during a predator encounter. Instead, you may often get alarm and recruitment calls together because it is common for individuals facing a threat to want their conspecifics to come help them.

Like the Japanese tits’ ‘ABC-D’ calls, sequences of signals that satisfy the requirements of compositional semantics might simply be composed of independent meaningful parts that co-occur in time and space, rather than being combined and working together in any significant way. The independent meaningful elements might well contribute to the meaning of the whole, but they could do so independently, rather than jointly. For example, it might not make any difference to the meaning of the sequences whether signals are produced by one or more individuals. Sequences of uncombined animal signals (in which signals function independently, rather than jointly) are too distant from the hierarchical structures of human syntax to be considered significantly continuous phenomena.

Despite this problem, there is some evidence that some of the sequences that satisfy the requirements of the compositional semantics framework may form actual combinations. For example, according to a recent study by Suzuki and Matsumoto (2022), it seems that it makes a difference to Japanese tits whether signals are produced by one or more individuals: they respond to ‘ABC-D’ sequences when they come from a single loudspeaker, but they do not respond when the alarm calls (‘ABC’) and the recruitment calls (‘D’) come from different loudspeakers, even though they are placed relatively close to one another (around 10 m). If Japanese tits perceived ‘ABC’ and ‘D’ as separate utterances, it should make no difference to them whether these calls are produced by two loudspeakers. The fact that Japanese tits fail to respond in the two loudspeakers scenario can be interpreted as evidence that they treat the ‘ABC-D’ sequences as combinations.

There is further empirical evidence that some animal sequences that satisfy the requirements of the compositional semantics framework may form genuine combinations. For example, Engesser et al. (2016, 2024) note that responses to alert-recruitment sequences in Southern pied babblers are ‘non-additive’, in that they are more than just the sum of the responses to the individual calls in the sequences. While responses to recruitment calls are typically characterised by receivers slowly approaching the caller, and alert calls are often ignored, when they hear alert-recruitment sequences, receivers become more attentive and approach the loudspeaker more rapidly than when they hear recruitment calls alone. Stronger evidence that at least some non-human animals are sensitive to combinations can be found in studies of enculturated great apes. In a detailed study, Savage-Rumbaugh et al. (1993) report that Kanzi the bonobo shows the ability to track the difference between sentences like “put oil in tomato” vs “put tomato in oil”. This suggests that Kanzi treats the sequence as a combination, and that he is sensitive to the linear order in which words appear in each sequence (for discussion see Truswell 2017. Note, however, that Truswell observes some limitations in Kanzi’s abilities that suggest he struggles with processing hierarchical syntax; on this, see also “Vindicating the possibility of a bounded hierarchy” section).

Evidence that certain animal sequences can be interpreted as proper combinations is encouraging as it suggests that human syntax may have roots in certain animal capacities. However, there is a second problem with the compositional semantics framework: even when animal sequences form actual combinations, they may not be hierarchically structured. To borrow from Jackendoff (2002), the signals composing animal sequences may be flatly concatenated rather than being processed as constituents, as parts of larger wholes (see also Progovac 2015). It remains to be seen how focusing on these non-hierarchical combinations can illuminate the emergence of hierarchical structure. If we accept that human syntax is hierarchical, then a satisfactory account of the evolution of syntax should also address the evolution of hierarchy. On this point, the compositional semantics framework seems inadequate.

## Toward a bounded hierarchy framework

In the previous sections, I argued that both the compositional semantics and unbounded hierarchy frameworks have problems when it comes to explaining the evolution of human syntax. The unbounded hierarchy framework is not warranted in requiring unboundedness (UH.2) for syntactic continuity; it is furthermore unjustified in establishing a discontinuity between animal sequences and human syntax on the basis of this requirement. Requiring unboundedness, the unbounded hierarchy framework sets too high of a bar for syntactic continuity. On the one hand, it requires empirical evidence of unbounded competence, which we do not possess even in the case of humans; on the other, it is unjustified in ruling out that precursors of an unbounded capacity could be found in bounded capacities. As a result, the unbounded hierarchy framework does not make a compelling case for a saltationist account of the evolution of human syntax. It discounts possible continuities between animal capacities and human syntax, as well as possible incremental stages in the development of human syntactic complexity.

Conversely, by not requiring hierarchy, the compositional semantics framework sets the bar for syntactic continuity too low: sequences that satisfy the criteria for compositional semantics (CS.1 and CS.2) may not involve genuine combinations of signals, and even when they do, these combinations may lack hierarchical structure. Failing to explain how hierarchical human syntax could have evolved from potentially unstructured sequences of signals, the compositional semantics framework does not provide a satisfactory incrementalist account of the evolution of human syntax.

The unbounded hierarchy framework is unwarranted in sacrificing syntactic continuity for unboundedness; the compositional semantics framework establishes syntactic continuity but at the cost of sacrificing hierarchy. It is possible to construct an alternative framework for studying the evolution of human syntax that avoids both these sacrifices. I call this the “bounded hierarchy framework”. In contrast to the unbounded hierarchy framework, the bounded hierarchy framework pursues the possibility that precursors of an unbounded capacity could be found in bounded capacities. In contrast to the compositional semantics framework, the bounded hierarchy framework directly addresses the problem of the evolution of hierarchy in human syntax.

Within the bounded hierarchy framework, for animal sequences to be regarded as continuous with human syntax, they must fulfil two basic requirements:

(BH.1) *Compositionality*: The sequence must be composed of individual units.

(BH.2) *Bounded hierarchy:* The sequence must exhibit a part-whole, hierarchical structure, with certain units grouped together and contained within larger structures (and this does not require unboundedness).

In contrast to the compositional semantics framework, requirements BH.1 and BH.2 are not restricted to the context of meaningful communication but can potentially be met by sequences in other domains as well. In the bounded hierarchy framework that I propose, units need not be, although can be, meaningful. This is because I take it that syntactic properties could also be expressed in contexts other than communication––for instance, in action, mathematics and music (see e.g. Mukherji 2022)––and that, accordingly, evolutionary precursors of syntax in communication could be found in domains other than communication (see e.g. Planer and Sterelny 2021 for an account of the evolution of human syntax from the cognitive capacities involved in toolmaking). It is possible, after all, to envisage an evolutionary scenario in which hierarchical abilities in non-communicative domains are exapted for communication, via processes that connect existing semantic and pragmatic communicative abilities with hierarchy. However, in the context of meaningful communication, requirements BH.1 and BH.2 could be satisfied as follows:

(BH.1) *Compositional semantics*: The sequence is composed of meaningful units, and its meaning is a function of the meanings of the individual signals.

(BH.2) *Bounded hierarchy:* The meaning of the whole sequence is a function of the hierarchical relations between the signals.

## Vindicating the possibility of a bounded hierarchy

Within the bounded hierarchy framework, animal sequences that turn out to be hierarchical can be considered continuous with human syntax and plausible evolutionary precursors even if they are not the product of an unbounded combinatorial capacity. The possibility of a bounded hierarchy can be vindicated both theoretically and empirically. Theoretically, there is no principled reason for thinking that unboundedness and hierarchy must come together, as proponents of the Merge hypothesis have it. Indeed, there is no conceptual dependence between the two: it is possible to think of hierarchy as separate from unboundedness. Empirically, a growing body of evidence suggests that nonhuman animals may possess limited forms of hierarchical cognition (Berwick et al. 2011, Byrne et al. 2013; Gontier et al. 2024; Watson et al. 2020, Lameira et al. 2024, Bosshard et al. 2024). One interesting case is studies of animal action.

On some views, the hierarchical structure found in natural language can also be found in some complex goal-directed actions (Lahsley 1951, Jackendoff 2002, Planer and Sterelny 2021, Planer 2023). Like natural language, some human actions exhibit a part-whole, hierarchical structure: an action such as making coffee is governed by a higher-order representation of the goal of making coffee, which organises and controls lower-order representations of subgoals, such as grinding beans, pouring water in the machine, activating the machine etc. (Planer 2023).^11^ Hierarchical models of human action stand in contrast to classic chain models, which conceive of behaviours as outcomes of reflex-like serial chains, in which each action is directly triggered or primed by the preceding action or its product. As Lashley (1951) observed, human actions are characterised by a level of flexibility that is incompatible with models of associative chaining: for example, people often omit or add steps in behavioural sequences (e.g. activating the coffee machine without having put coffee in it, pour water if there is none). They can also easily resume actions after interruption (e.g. returning to make coffee after answering a phone call). This would not be possible if the behaviours were completely chain-like: as argued by Fitch and Martins (2014), in behaviours where each action serves as the stimulus for the next one, flexibly inserting subactions is not easy, and omitting any steps would likely cause the sequence to stop. It is possible, instead, if, during the act of making coffee, the higher-level goal of making coffee remains activated while lower-level goals are executed (e.g. grinding beans), or when interruptions occur (e.g. a phone call; see Planer and Sterelny 2021).

While chain models long dominated accounts of animal behaviour, recent evidence suggests that some animal behaviours, too, exhibit a degree of flexibility that cannot be adequately described as purely linear or chain-like. In termite fishing, for example, chimpanzees both omit optional steps (e.g., skipping the step of puncturing a termite mound if a hole already exists) and embed additional steps into the behavioural sequence (e.g., pausing to adjust or swap tools before resuming their activity). According to scholars such as Byrne et al. (2013), flexible behaviours such as those of chimpanzees can be described in terms of hierarchically structured representations of goals and sub-goals like those of humans, where representations of higher-level goals (e.g. fishing termites) control lower-level goal representations and persist while the latter are carried out (see also Sanz et al. 2009 and Gontier 2024). Recent quantitative evidence further supports this interpretation. According to Howard-Spink et al. (2024), tool use behaviours in chimpanzees exhibit patterns of non-adjacent dependencies, in a way that is consistent with a hierarchical arrangement of sub-actions. While in behaviours that are chain-like the predictability of an action decreases as the temporal distance from another action increases, in chimpanzee tool use an action in a sequence can predict other actions that are temporally very distant. This suggests a hierarchical, rather than strictly linear, organisation of behaviour.

Evidence for hierarchical organization in chimpanzee action is also consistent with evidence of hierarchy in other cognitive domains and species. For example, some studies find that chimpanzees can recognise nonadjacent dependencies in both visual and auditory sequences (Sonnweber et al. 2015; Watson et al. 2020). As Berwick et al. (2011) observe, the songs of some birds exhibit a hierarchical structure, where individual notes can be combined into syllables, syllables into ‘motifs’, and motifs into complete song ‘bouts’ (see Sainburg et al. 2019 for further quantitative evidence). Recent work by Lameira et al. (2024) identifies some possible evidence of hierarchical organisation in orangutan long calls. Studies by Bayern et al. (2018) show that New Caledonian Crows can spontaneously construct compound tools composed of three or four parts to reach otherwise unreachable food, a behaviour that may be viewed as governed by hierarchically structured goal representations.

Of course, there are some reasons to be cautious in handling this evidence. Existing studies are relatively few, and it is possible to draw only limited conclusions about what they demonstrate. Indeed, there is also some evidence that animals find it difficult to process hierarchical structures. According to Truswell (2017), the bonobo Kanzi struggles with sentences involving noun phrase (NP) coordination like “Fetch the tomato and the oil”, whose interpretation requires grouping the words “tomato” and “oil” together, as constituents of a higher-order noun phrase. In Truswell’s view, Kanzi’s ability to understand other syntactically structured linguistic utterances in English is not necessarily indicative of hierarchical processing capacities. Understanding sentences like “Kanzi, could you cut the onions with your knife?” could in fact be achieved without hierarchical processing. For instance, Kanzi might infer the required action based on the individual meanings of the words and his experience in interacting with knives and onions in a certain way—a strategy Anderson (2004) has dubbed “semantic soup.” Furthermore, while Kanzi’s ability to track the difference between sentences like “Put the tomato in the oil” and “Put some oil in the tomato” could not be explained by a semantic soup strategy, it could be explained by positing that Kanzi is sensitive to the linear order in which words are produced.^12^

There is, moreover, a key methodological challenge in attributing hierarchical capacities to nonhuman animals. Hierarchical structures are mental constructs and must be inferred indirectly from observable sequential behaviour, both verbal and not verbal. However, not all sequential behaviours that can be described hierarchically are necessarily produced or governed by hierarchical processes. How can we ascertain that a hierarchical description tracks something real, that hierarchy has a psychological reality? This is particularly problematic as we humans are often biased towards hierarchy (Fitch 2014; Ferrigno et al. 2020).

While not conclusive, evidence of hierarchical cognition in animals might still have important implications for our understanding of the evolution of human syntax, which deserve to be explored. If bounded hierarchy is a theoretical and empirical possibility, the bounded hierarchy framework could help us explore the possibility that evolutionary precursors of human syntax could be found in simpler forms of bounded hierarchy that are also present in extant nonhuman animals.

In contrast to the unbounded hierarchy framework, the bounded hierarchy framework claims that syntax did not emerge fully formed as an unbounded system of hierarchical structure, but evolved incrementally from bounded, rather than unbounded, hierarchical capacities. In contrast to the compositional semantics framework, the bounded hierarchy framework can explain how we arrived at the complex and sophisticated hierarchical structures that characterise human syntax from simple forms of hierarchical cognition. The working hypothesis of the bounded hierarchy framework is that our syntactic capacity became increasingly more powerful as it was managed by a progressively enhanced cognitive system, driven by certain selective pressures (e.g. communication). It is plausible that human syntactic capacity was potentiated with the aid of other human cognitive capacities acting in concert, such as memory, attention and inhibitory control.

For example, domain-general capacities such as memory, attention and inhibitory control determine how many units can be concatenated in a sequence: an agent can only bind as many units as their memory can hold. Additionally, processing progressively unfolding strings of signs requires attentional focus on the task and the ability to inhibit other incoming stimuli. Furthermore, being able to represent things in a way that is not limited to immediate circumstances is certainly key to producing a wide range of utterances here and now, including communicating about things in different spaces and times. Even proponents of the Merge hypothesis accept that the power of human syntax is partly due to the fact that Merge operates on symbols, that is representational items that can be tokened independently of specific stimuli or contexts (Bickerton 2014). The largely symbolic (i.e. stimulus-independent) nature of human communication can be generated by some of the capacities mentioned above, including memory, attention, inhibitory control, as well as by other capacities such as range of possible goals, and the very fact of having a language system (see e.g. Millikan 2017).

## Conclusion

In this paper, I have reconstructed the “unbounded hierarchy” (Bolhuis et al. 2018) and the “compositional semantics” (Townsend et al. 2018) frameworks for studying the evolution of human syntax. I have argued that, in requiring unboundedness, the unbounded hierarchy framework requires too much for syntactic continuity, while, in sacrificing hierarchy, the compositional semantics framework does not require enough to provide a satisfactory account of the evolution of human syntax. Considering these problems as well as empirical evidence of hierarchy in nonhuman animals, I have provided my “bounded hierarchy” framework for the study of the evolution of human syntax. The bounded hierarchy framework that I propose traces the evolutionary origins of human syntax to simpler forms of bounded hierarchy that may be present in extant nonhuman animals. This framework can offer an alternative account of the evolution of human syntax, one that is incrementalist but more complete than the incrementalist account provided by the compositional semantics framework. In contrast to the saltationist account of the evolution of human syntax provided by the unbounded hierarchy framework, the bounded hierarchy framework does not exclude the possibility that human syntax could have evolved from bounded capacities also present in extant nonhuman animals. In contrast to the incrementalist account provided by the compositional semantics framework, the bounded hierarchy framework can tell a more complete story of the evolution of human syntax, directly connecting evidence of hierarchy in the animal kingdom to the complex and sophisticated hierarchical structures found in language.

## Footnotes

1 Schlenker et al. (2023)’s “one-utterance hypothesis” is a deflationary alternative to theories of animal compositionality. The hypothesis proposes that sequences that appear combinatorial may, in some cases, be single signals that are just acoustically more complex than others.

2 In contrast to Suzuki et al. (2018), Bolhuis et al. (2018) argue that it is not obvious that Japanese tits respond less to artificially reversed ‘D-ABC’ sequences because they are sensitive to linear order. This could also be because they are only able to respond to signals that are present in their natural repertoire. Bolhuis et al.’s interpretation, however, contrasts with findings showing that Japanese tits are in fact able to respond to artificial sequences (see e.g. the case of the ‘ABC-tää’ sequences). Moreover, it is worth noting that the results in Suzuki et al. (2018)’s study have not been replicated in other studies of animal sequences. For example, recent findings from Engesser et al. (2024) show that Southern pied babblers respond to artificially reversed ‘recruitment-alert’ sequences in the same way they respond to their natural alert-recruitment sequences.

3 Linguistic analyses of constituent structure typically also include information about grammatical categories. Words, for example, can belong to grammatical categories like nouns, verbs and adjectives. Phrases, in turn, are denoted by grammatical categories based on the category of their head (i.e. the word of which they are projection from). For example, a noun phrase is a phrase headed by a noun (e.g. “The white cat”), while a verb phrase is a phrase headed by a verb (e.g. “ran away”) (see Jackendoff 2002). The syntactic category to which a lexical item or phrase belongs can determine the rules by which it may combine with other elements. While grammatical categories and headedness are important aspects of natural language syntax, in this paper I will focus solely on hierarchical structure.

4 It is worth noting that, for proponents of unbounded hierarchy, Merge is an instrument of thought, not just of communication (see e.g. Berwick and Chomsky 2016), and can be expressed also in other contexts, such as music, action and mathematics (e.g. Mukherji 2022; see also “Toward a bounded hierarchy framework” section).

5 Note that, unlike unbounded hierarchy, proponents of compositional semantics, like Townsend et al. (2018), only focus on syntactic properties in meaningful communication. Given requirements CS.1 and CS.2, the compositional semantics framework excludes two main categories of animal signal sequences: phonological syntax and combinatorial syntax (Hurford 2011). Phonological syntax refers to cases of animal sequences, in which elements are arranged according to specific patterns but do not seem to be individually meaningful (e.g. the songs of Bengalese finches and Nightingales, Berwick et al. 2011). Combinatorial syntax refers to cases of animal sequences in which elements are individually meaningful, but in which the meaning of the whole is not a function of the meanings of its parts, such as the ‘pyow-hack’ sequences of putty-nosed monkeys. As noted by Arnold & Zuberbühler (2012), putty-nosed monkeys’ ‘pyow-hack’ sequences are composed of individually meaningful signals—‘pyows’ (alert calls) and ‘hacks’ (recruitment calls)—but since the sequence as a whole elicits group travel, it is unlikely that its meaning is derived from the individual meanings of ‘pyows’ and ‘hacks’ (see Schlenker et al. 2016 for a different view).

6 The dominant view is that the computational operation Merge was selected for thinking and planning, rather than communication (see e.g. Bickerton 2014 and Berwick and Chomsky 2016).

7 Note that language is hierarchical also below the sentence level: below the sentence level, words are composed of morphemes, which are themselves composed of phonemes. However, see Frank et al. (2012) for a view that hierarchical processing is less pervasive in language than commonly thought.

8 It’s worth noting is that, while proponents of the unbounded hierarchy framework recognise that hierarchy is central to human syntax, they haven’t really done much to explain it, apart from positing that it results from Merge. How hierarchy actually works is an important question that I will set out to address in a separate paper.

9 The material on the competence-performance distinction was originally drafted as part of a co-authored SEP entry with Richard Moore (Moore and Palazzolo 2024) but was ultimately cut from the final version. It therefore has its origins in joint work.

10 It is possible to imagine a single combinatorial mechanism that is more powerful in human beings because it is potentiated by the greater complexity and sophistication of their cognitive system, and less powerful in animals because constrained by their limited cognitive capacities.

11 Moro (2014) and Coopmans et al. (2022) have argued against the claim that action is hierarchically structured in a similar sense to natural language. They identify some differences that, in their view, make this parallelism unsuitable. For example, Moro points out that the atomic units manipulated by syntax and action are fundamentally different. Actions do not have functional elements like ‘the’, ‘if’ and ‘not’. Furthermore, much of the structure of action is shaped by external physical constraint, which is not the case of natural language. Only in natural language, moreover, locality conditions constraint possible syntactic combinations. Coopmans et al. (2022) highlight that while a same action can be accomplished by different hierarchical structures, in natural language differences in hierarchical structure systematically correspond to differences in meaning. However, it is important to note, none of the critiques raised by Moro (2014) and Coopmans et al. (2022) challenge the view that natural language and action both exhibit a part-whole, hierarchical structure. What these critiques show, instead, is that the parallelism is not perfect and some differences exist. As argued by Boeckx and Fujita (2014), from an evolutionary point of view differences in hierarchical processing across domains are to be expected, as cognitive capacities can undergo modifications and acquire novel features in certain domains and when instantiated by different cognitive systems.

12 A pioneering study by Herman et al. (1984) suggests that bottlenose dolphins can track relations that go beyond linear word order. In this study, the dolphin Akeakamai demonstrates some ability to master a non-linear grammar. While in a linear grammar the meaning of a sentence is encoded by the left-to-right order of the elements, in a nonlinear grammar the indirect object precedes the direct object. For example, an instruction such as “go to the hoop and take it to the pipe” is expressed as “pipe fetch hoop” in a linear grammar, as “hoop fetch pipe” in a nonlinear grammar. Although it remains to be seen whether the ability to master a nonlinear grammar in this task can constitute evidence for hierarchical processing, Akeakamai’s performance surpasses what has been reported in the case of Kanzi. Simply following the sequential order of words is in fact not sufficient to correctly interpret the non-linear grammar instruction.
